# Myocyte-derived Tnfsf14 is a survival factor necessary for myoblast differentiation and skeletal muscle regeneration

**DOI:** 10.1038/cddis.2015.375

**Published:** 2015-12-31

**Authors:** R J Waldemer-Streyer, J Chen

**Affiliations:** 1 Department of Cell and Developmental Biology, University of Illinois at Urbana-Champaign, Urbana, IL, USA

## Abstract

Adult skeletal muscle tissue has a uniquely robust capacity for regeneration, which gradually declines with aging or is compromised in muscle diseases. The cellular mechanisms regulating adult myogenesis remain incompletely understood. Here we identify the cytokine tumor necrosis factor superfamily member 14 (Tnfsf14) as a positive regulator of myoblast differentiation in culture and muscle regeneration *in vivo*. We find that Tnfsf14, as well as its cognate receptors herpes virus entry mediator (HVEM) and lymphotoxin *β* receptor (LT*β*R), are expressed in both differentiating myocytes and regenerating myofibers. Depletion of Tnfsf14 or either receptor inhibits myoblast differentiation and promotes apoptosis. Our results also suggest that Tnfsf14 regulates myogenesis by supporting cell survival and maintaining a sufficient pool of cells for fusion. In addition, we show that Akt mediates the survival and myogenic function of Tnfsf14. Importantly, local knockdown of Tnfsf14 is found to impair injury-induced muscle regeneration in a mouse model, affirming an important physiological role for Tnfsf14 in myogenesis *in vivo*. Furthermore, we demonstrate that localized overexpression of Tnfsf14 potently enhances muscle regeneration, and that this regenerative capacity of Tnfsf14 is dependent on Akt signaling. Taken together, our findings reveal a novel regulator of skeletal myogenesis and implicate Tnfsf14 in future therapeutic development.

Mature skeletal muscle tissue contains a resident population of stem cells that imparts a great capacity for regeneration. Upon injury, these quiescent satellite cells are reactivated and begin to proliferate.^1, 2^ Effective myogenesis depends on the daughter myoblasts successfully differentiating and fusing with each other to regenerate the characteristic multinucleated skeletal myofibers. This involves a number of highly regulated steps, including activation of myogenic genes, migration, cell–cell adhesion and alignment, and finally membrane fusion.^3, 4, 5^ The fundamental principles underlying each step are well-conserved across species.^6^ Pathologies may result from dysregulation of these processes, including the suite of muscular dystrophies, cachexia and sarcopenia. However, the complex signaling mechanisms underlying skeletal myogenesis are still not fully understood.

It has long been accepted that the secreted factors influencing muscle cell regeneration *in vivo* are largely of immune cell origin; indeed, immune cells have been reported to reach concentrations over 100 000 cells/mm^3^ in regenerating muscle tissue.^7^ Recently, however, muscle cells are being revealed as prolific secretors of a wide variety of cytokines and growth factors,^8, 9, 10, 11^ including several that attract immune cells to regenerating muscle.^7^ Secretome studies show that myoblasts secrete different factors during proliferation than during differentiation, and even at different time points throughout differentiation.^8, 10, 11^ Another study identified numerous chemokine mRNAs expressed by differentiating myoblasts, which may be involved in regulating cell migration during myogenesis.^9^ However, functions of the newly identified muscle-secreted cytokines are mostly unexplored. Using RNAi, we conducted the first functional screen of cytokines for their impact on myogenic differentiation in C2C12 myoblasts, which allowed us to identify potential regulators of myogenesis in distinct functional groups.^12^ These results suggest the intriguing possibility that muscle cell-secreted proteins have a previously under-appreciated role in modulating muscle development and regeneration.

The function of cytokines in myogenesis is relevant to our understanding of not only basic muscle physiology, but also the diseases that negatively affect the health of muscle tissue, such as cachexia. Cachexia is characterized by extreme wasting of lean body mass and often occurs with an underlying chronic illness, such as cancer or congestive cardiac failure.^13^ Muscle atrophy during cachexic states ultimately stems from ubiquitin-mediated breakdown of myofibrils.^14^ Significantly, a well-documented association exists between cachexia and the dysregulation of cytokines, most notably the pro-inflammatory cytokines tumor necrosis factor alpha (TNF*α*), interleukin-1 (IL-1) and interleukin-6 (IL-6).^14, 15^

Tumor necrosis factor superfamily member 14 (Tnfsf14), also known as LIGHT (homologous to lymphotoxins, shows inducible expression, and competes with herpes simplex virus glycoprotein D for herpes virus entry mediator (HVEM), a receptor expressed by T lymphocytes), exists in two main forms: a type II transmembrane glycoprotein that projects extracellularly, and a soluble cytokine formed by cleavage of the extracellular portion of the protein off of the cell membrane.^16^ Through its receptors in the TNF receptor (TNFR) superfamily, HVEM (TNFRSF14) and lymphotoxin *β* receptor (LT*β*R), Tnfsf14 signaling is involved in lymphoid organ development and organization, as well as innate and adaptive immune responses.^17, 18, 19^ In recent years, Tnfsf14 has also emerged as a promising candidate for cancer immunotherapy.^20^

Tnfsf14 regulates cell survival and apoptosis in lymphocytes and tumor cells, and the cellular context determines whether Tnfsf14 is pro-survival or pro-apoptosis.^20, 21, 22^ Neither the expression nor the function of Tnfsf14 or its receptors has been reported in skeletal muscles. Our current study uncovers Tnfsf14 as a critical regulator of myoblast differentiation and muscle regeneration by governing myoblast survival, and implicates Tnfsf14 in potential therapeutic development for maintenance of muscle health.

## Results

### Tnfsf14 is required for myoblast differentiation

**Figure 1:**
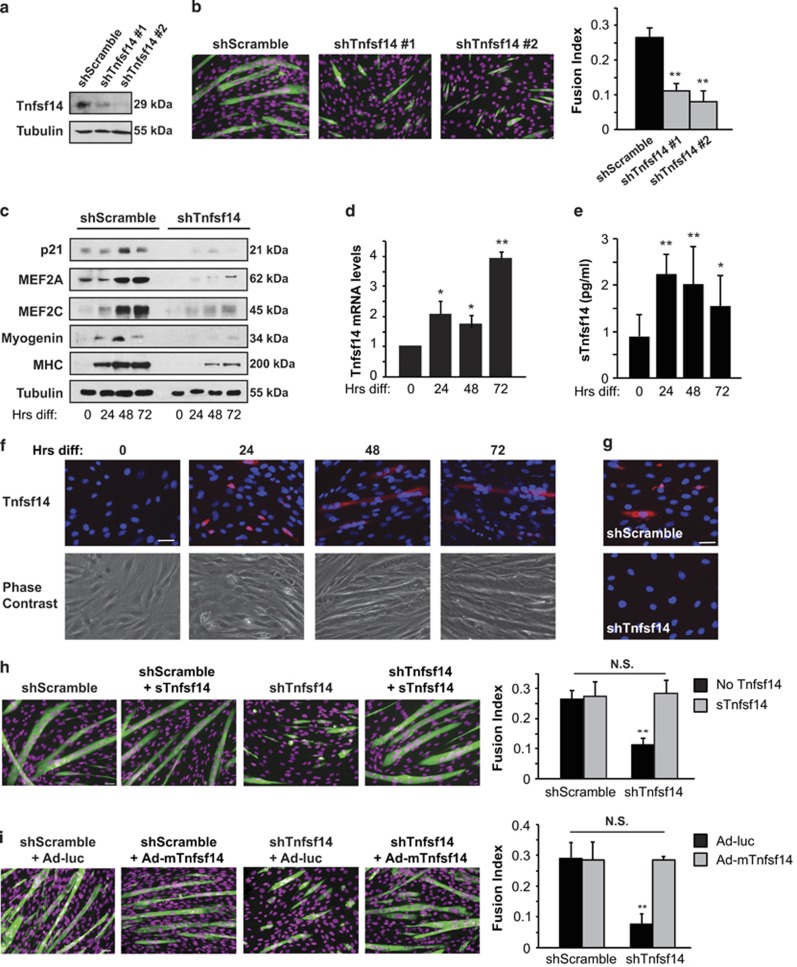
Tnfsf14 is a positive regulator of myoblast differentiation. (**a**) C2C12 cells were infected with lentiviruses expressing shTnfsf14 or shScramble (negative control), selected for 2 days, followed by cell lysis and western analysis (*n*=4). (**b**) Cells treated as in (**a**) were differentiated for 72 h, followed by staining for MHC (green) and DAPI (pseudo-colored magenta), and quantification of fusion index (*n*=5). (**c**) Cells treated as in (**a**) were differentiated, and at indicated time points ('Hrs diff') were lysed and subjected to western analysis (*n*=4). (**d**) RNA was isolated from differentiating C2C12 cells at indicated time points and subjected to qRT-PCR analysis for Tnfsf14 mRNA levels (*n*=6). (**e**) Cell media over the course of differentiation were subjected to ELISA assay to determine sTnfsf14 levels (*n*=8). (**f**) Differentiating C2C12 cells at various time points ('Hrs diff') were stained without permeabilization for Tnfsf14 (red) and DAPI (blue) (*n*=4). (**g**) Cells treated as in **a** were differentiated for 24 h, followed by staining for Tnfsf14 (red) and DAPI (blue) (*n*=3). (**h**) C2C12 cells were treated as in (**a**), and then differentiated in the presence or absence of 25 ng/ml recombinant sTnfsf14 for 3 days, followed by staining for MHC and DAPI, and quantification of fusion index (*n*=3). (**i**) C2C12 cells were treated as in (**a**), and then infected with adenoviruses expressing mTnfsf14 or luciferease (luc; negative control), followed by differentiation for 3 days and then staining for MHC and DAPI. The fusion index was quantified (*n*=3). All error bars represent S.D. of independent replicates. One-sample two-tailed *t*-test was performed for data in (**d**), and paired two-tailed *t-*tests for all other data. *P<0.05; **P<0.01. Scale bars: 50 *μ*m

The mouse myoblast cell line C2C12 offers a convenient *in vitro* system to study myoblast differentiation, as well as the effects of gene knockdown by lentivirus-delivered shRNA. We found that knockdown of Tnfsf14 (Figure 1a) significantly impaired C2C12 myotube formation, as indicated by myosin heavy chain (MHC) staining of the myocytes and quantification of the fusion index (Figure 1b). Two independent shRNAs yielded similar results, confirming the specificity of RNAi targeting. Moreover, Tnfsf14 depletion reduced the expression of muscle differentiation-specific proteins (Figure 1c), including the early myogenic markers MEF2A, p21 and myogenin. This result suggests that Tnfsf14 functions during the early stages of differentiation.

We set out to examine the expression and function of Tnfsf14 in myoblasts. As shown in Figure 1d, we found that Tnfsf14 expression increased over the course of differentiation at the mRNA level. The amount of the soluble cytokine (sTnfsf14) detected in the media by ELISA also increased significantly during differentiation (Figure 1e), although the concentrations were only at the lowest detection threshold of the assay. To probe the membrane-bound form (mTnfsf14), we immunofluorescently labeled differentiating myoblasts that had been washed to remove sTnfsf14, but that were not permeabilized, thus ensuring that all signal came from the external side of the cells. We found little mTnfsf14 signal in proliferating myoblasts, but observed mTnfsf14 expression in differentiating myocytes at 24 h, as well as myotubes at later time points of differentiation (Figure 1f). Tnfsf14 knockdown eliminated the immunofluorescence signal (Figure 1g), confirming the specificity of immunostaining. It is possible that mTnfsf14 is the predominant form of this cytokine in muscle cells. Nevertheless, the addition of recombinant sTnfsf14 to the differentiation media rescued myotube formation in cells depleted of Tnfsf14 (Figure 1h). A constitutively membrane-bound form of recombinant Tnfsf14 delivered by adenovirus ^23^ also fully reversed Tnfsf14 knockdown phenotype (Figure 1i). Hence, the two forms of Tnfsf14 appeared to function similarly in myocytes. The recombinant proteins did not enhance differentiation of cells without Tnfsf14 depletion (Figures 1h and i); endogenous Tnfsf14 may not be limiting. Tnfsf14 knockdown in mouse primary myoblasts also led to impaired myotube formation, which was rescued by adding recombinant sTnfsf14 protein to the media (Supplementary Figure S1). Taken together, these observations strongly suggest that Tnfsf14 has a positive cell-autonomous role in skeletal muscle differentiation.

### Tnfsf14 is necessary for maintaining sufficient cell numbers for differentiation

**Figure 2:**
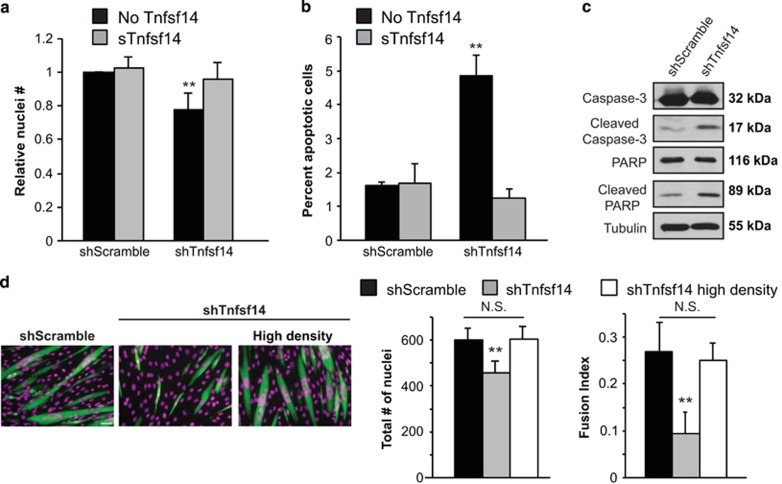
Tnfsf14 maintains sufficient cell numbers for differentiation. (**a**) C2C12 cells were infected with lentiviruses expressing shTnfsf14 or shScramble, selected for 2 days, followed by differentiation for 72 h in the presence or absence of 25 ng/ml recombinant sTnfsf14 and subsequent staining with DAPI. Stained nuclei were counted (*n*=3). (**b**) Cells were treated as in (**a**) but differentiated for 24 h, followed by TUNEL assay to detect apoptotic cells (*n*=4). (**c**) Primary myoblasts were infected with shRNA lentiviruses and differentiated for 24 h, followed by cell lysis and western analysis (*n*=3). (**d**) C2C12 cells were infected with shRNA lentiviruses and then plated at different densities in order to compensate for the lower cell number seen in Tnfsf14 knockdown cells. Cells were differentiated for 72 h and subsequently stained for MHC. Nuclei number and fusion index were quantified (*n*=4). Scale bar: 50 *μ*m. One-sample two-tailed *t-*test was performed for data in (**a**), and paired two-tailed *t*-tests for all other data. **P<0.01. All error bars represent S.D. of independent replicates

In addition to inhibiting myotube formation, knockdown of Tnfsf14 also reduced the number of cells present in culture over the course of differentiation (Figure 2a). Importantly, this decrease in cell number was corrected by adding sTnfsf14 to differentiating cells (Figure 2a). We wondered whether the lower number of cells upon Tnfsf14 knockdown was a result of reduced survival in this cell population. Indeed, Tnfsf14 depletion increased apoptosis in differentiating myoblasts, as indicated by a markedly increased incidence of TUNEL labeling (Figure 2b), as well as elevated caspase-3 activation and PARP cleavage (Figure 2c). Addition of sTnfsf14 in Tnfsf14-depleted cells reversed the cell death rate to normal levels (Figure 2b). Interestingly, we found that Tnfsf14 could also function as a survival factor in proliferating myoblasts exposed to etoposide, which is known to induce apoptosis in C2C12 cells.^24^ As shown in Supplementary Figure S2A, sTnfsf14 drastically reduced the apoptosis rate in etoposide-treated cells. Hence, the function of Tnfsf14 in supporting myoblast survival is not limited to the differentiation process. Notably, cell proliferation as measured by bromodeoxyuridine (BrdU) labeling was also diminished by Tnfsf14 knockdown 24 h after the onset of differentiation; BrdU labeling could be restored to normal levels by recombinant sTnfsf14 (Supplementary Figure S2B). However, since by 24 h of differentiation myoblasts have largely withdrawn from the cell cycle, the further reduction of proliferation is unlikely to contribute significantly to the reduced cell number in Tnfsf14-depleted cells. Instead, loss of cell survival is likely responsible.

In light of the results above, we next asked whether the impaired differentiation phenotype upon Tnfsf14 knockdown was a direct consequence of reduced cell number. To probe this question, we plated C2C12 cells depleted of Tnfsf14 at increasing densities, thus normalizing cell numbers to those of the control by the end of differentiation. Remarkably, we found that Tnfsf14 knockdown cells differentiated normally when the cell numbers were brought up to control levels (Figure 2d). This observation suggests that Tnfsf14 promotes myogenesis by maintaining a sufficient number of myoblasts in the differentiating population.

### HVEM and LT*β*R, receptors for Tnfsf14, are required for myoblast differentiation and survival

**Figure 3:**
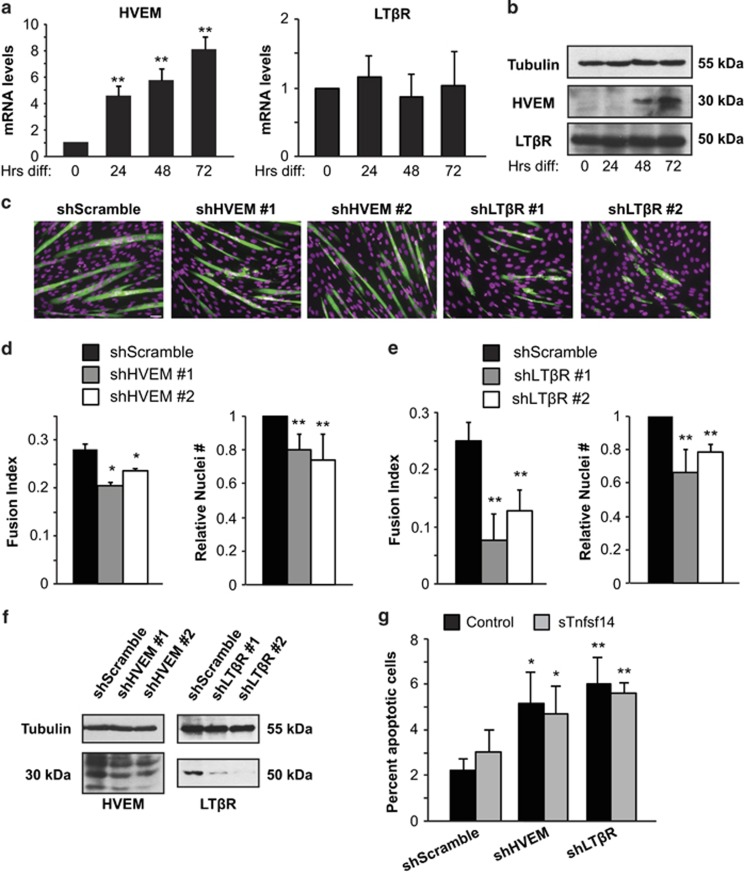
Both HVEM and LT*β*R are required for myoblast differentiation and survival. (**a**) HVEM or LT*β*R mRNA transcripts were measured over the course of C2C12 differentiation by qRT-PCR (*n*=4 for HVEM and *n*=3 for LT*β*R). (**b**) HVEM and LT*β*R protein levels during C2C12 cell differentiation were analyzed by western blotting (*n*=4). (**c**) C2C12 cells were infected with shRNA lentiviruses as indicated, selected for 2 days, followed by differentiation for 72 h and subsequent staining for MHC and with DAPI. Scale bar: 50 *μ*m. (**d** and **e**) Fusion index and total nuclei number were quantified for experiments in (**c**) (*n*=5). (**f**) Lentivirus-mediated knockdown of HVEM and LT*β*R in C2C12 cells were confirmed by western analysis (*n*=3 for HVEM and *n*=4 for LT*β*R). (**g**) C2C12 cells treated as in (**c**) but differentiated for 24 h were subjected to TUNEL assays, and the percentage of apoptotic cells was measured (*n*=4). One-sample two-tailed *t*-test was performed for data in (**a**) and relative nuclei number in (**d**) and (**e**), paired two-tailed *t*-tests for all other data. *P<0.05; **P<0.01. All error bars represent S.D. of independent replicates

HVEM and LT*β*R are established receptors for Tnfsf14 in various types of cells.^18^ To assess whether Tnfsf14 would also signal through one or both of these receptors in skeletal muscle, we first examined the expression of these two receptors in muscle cells. Indeed, we found both receptors to be expressed in C2C12 cells. HVEM expression was drastically upregulated over the course of differentiation at both the mRNA and protein levels (Figures 3a and b), whereas LT*β*R levels were not significantly changed (Figures 3a and b). Importantly, knockdown of either HVEM or LT*β*R by lentivirus-delivered shRNA led to a similar phenotype to that of Tnfsf14 knockdown: impaired myotube formation and a lower cell number (Figures 3c–e). The effects were observed with two independent shRNAs (Figure 3f). We did not observe an additive effect on myogenesis or cell number when both receptors were depleted simultaneously, although knockdown efficiency for each gene was typically inferior when the cells were co-infected by two shRNA viruses targeting two separate genes (data not shown). Collectively, these data suggest that both HVEM and LT*β*R may function as the myogenic receptors of Tnfsf14 in a non-redundant manner.

To further validate the roles of HVEM and LT*β*R in mediating the action of Tnfsf14, we examined the effect of depleting the receptors on cell survival. We observed increased TUNEL signal in C2C12 cells depleted of either receptor (Figure 3g). Notably, this increased cell death was not reversed by the addition of sTnfsf14. This is in contrast to the impaired survival phenotype seen in cells depleted of Tnfsf14, which could be rescued by the recombinant protein, and is consistent with Tnfsf14 acting through these receptors. These observations suggest that both HVEM and LT*β*R may mediate Tnfsf14 function in cell survival. We also measured BrdU labeling in C2C12 cells with HVEM or LT*β*R knockdown. A lower fraction of BrdU-positive cells was observed upon the depletion of LT*β*R, but not of HVEM (Supplementary Figure S3), reminiscent of Tnfsf14 knockdown (Supplementary Figure S2A). Hence, both LT*β*R and HVEM are involved in cell survival, whereas only LT*β*R has a role in cell proliferation.

### Akt mediates Tnfsf14 signaling

**Figure 4:**
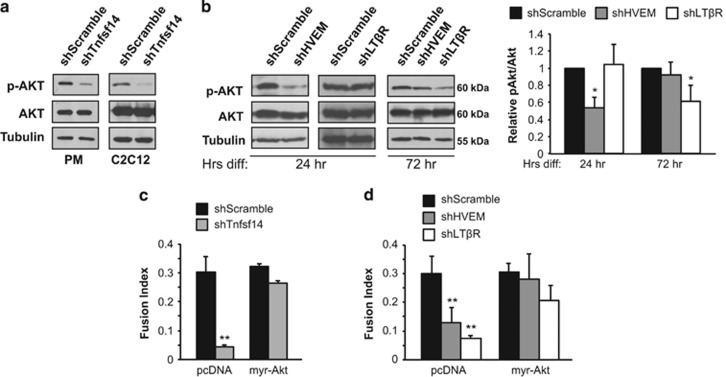
Tnfsf14 regulates myoblast differentiation through Akt. (**a**) Primary mouse myoblasts (*n*=4) and C2C12 cells (*n*=5) were infected with shRNA lentiviruses and differentiated for 24 h, followed by cell lysis and western analysis. (**b**) C2C12 cells were infected with shRNA lentiviruses as indicated and differentiated for 24 or 72 h, followed by cell lysis and western analysis (*n*=5). (**c** and **d**) C2C12 cells were infected with shRNA lentiviruses as indicated, and then transfected with a constitutively active Akt or a control (pcDNA) construct, followed by differentiation for 72 h and subsequent staining for MHC and with DAPI. Fusion index and total nuclei number were quantified (*n*=4). One-sample two-tailed *t*-test was performed for data in (**b**) and paired two-tailed *t*-tests for data in (**c**) and (**d**). *P<0.05; **P<0.01. All error bars represent S.D. of independent replicates

We next considered the molecular pathway that mediates Tnfsf14's myogenic function. One of the most attractive candidates was Akt, which is a known mediator of cell survival and proliferation.^25, 26^ Akt is also a key regulator of myogenic differentiation^27^ and muscle mass,^28^ and its role in myoblast survival has been reported.^29, 30^ In addition, HVEM signaling has been previously linked to Akt activation in T cells.^31^ Indeed, we observed decreased levels of phosphorylated Akt in both primary myoblasts and C2C12 cells depleted of Tnfsf14 (Figure 4a). Knockdown of HVEM also impaired Akt phosphorylation during the early stages of differentiation, whereas LT*β*R depletion had a similar effect at a later time of differentiation (Figure 4b). Therefore, HVEM and LT*β*R may both signal through Akt, but in a temporally regulated manner. To assess the functional relevance of Akt in Tnfsf14 myogenic signaling, we expressed a constitutively active (c.a.) Akt^32^ in C2C12 cells. As shown in Figure 4c, the impaired differentiation observed after Tnfsf14 knockdown was fully rescued by the expression of c.a.-Akt. Notably, c.a.-Akt also rescued differentiation in myoblasts depleted of HVEM or LT*β*R, providing further evidence that both receptors signal independently through Akt. Taken together, our observations strongly suggest that Tnfsf14 regulates myoblast differentiation via Akt.

### Tnfsf14 is necessary for robust skeletal muscle regeneration

**Figure 5:**
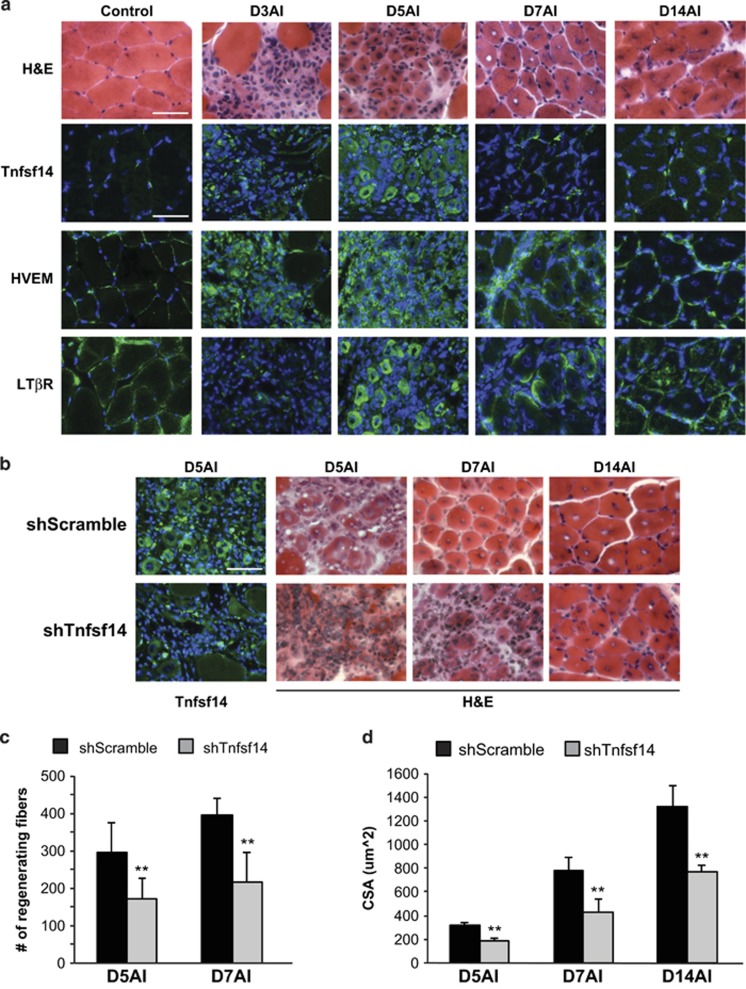
Tnfsf14 is required for robust muscle regeneration *in vivo*. (**a**) TA muscles were injured by BaCl_2_ injection, and isolated on days 3, 5, 7 and 14 AI. Upon cryosection, H&E staining or immunofluorescence staining for Tnfsf14, HVEM and LT*β*R (green) together with DAPI (blue) were performed (*n*=3). (**b**) TA muscles were co-injected with BaCl_2_ and shRNA viruses, and then processed as described in (**a**). (**c**) Quantification of the number of regenerating myofibers on muscle sections from (**b**). (**d**) Quantification of the regenerating myofiber cross-sectional area (CSA) on muscle sections from (**b**). For each time point in (**b**–**d**), five or six mice were analyzed. All error bars represent S.D. of independent replicates. Paired two-tailed *t*-test was performed to compare shScramble and shTnfsf14 at each time point. **P<0.01. Scale bars: 50 *μ*m

In order to probe the physiological relevance of Tnfsf14's myogenic function, we utilized a well-established murine model of post-injury skeletal muscle regeneration.^33, 34^ Barium chloride (BaCl_2_) was injected into the tibialis anterior (TA) muscle of the hindlimb to induce localized necrosis. As shown in Supplementary Figure S4, Tnfsf14 mRNA levels measured by qRT-PCR rose on day 3 after injury (AI) and returned to basal levels by day 5 AI (Supplementary Figure S4A). As multiple cell types present in regenerating muscle could contribute to this Tnfsf14 mRNA expression, we performed immunohistochemistry on sections of regenerating TA muscles. As shown in Figure 5a, no Tnfsf14 expression was detected in undamaged muscle, but Tnfsf14 staining was noted in mononucleated cells on day 3 AI. To determine whether these Tnfsf14-positive cells were myogenic, we co-labeled injured muscles on day 3 AI for both Tnfsf14 and MyoD, a marker of activated and proliferating satellite cells. As shown in Supplementary Figure S4B, Tnfsf14+/MyoD+ cells were observed, demonstrating that myogenic progenitors express Tnfsf14 during the early phase of regeneration. We also observed Tnfsf14+/MyoD– cells, which could represent either infiltrating immune cells or myogenic progenitors not yet expressing MyoD. Strikingly, strong Tnfsf14 staining was also clearly observed in the newly formed myofibers visible on day 5 AI (Figure 5a), unequivocally revealing the expression of Tnfsf14 by regenerating muscle cells.

We also performed immunohistochemistry to examine the two Tnfsf14 receptors in muscle as shown in Figure 5a. Unlike Tnfsf14, HVEM and LT*β*R were both found at the periphery of myofibers in uninjured muscles. Interestingly, most mononucleated cells in the early stages of regeneration exhibited a strong HVEM signal, whereas LT*β*R signal was only weakly present in a small number of mononucleated cells. Newly regenerated myofibers on day 5 AI stained strongly for LT*β*R and were also positive for HVEM, although the latter continued to be predominantly expressed in mononucleated cells. By day 7 AI, both receptors were found at the periphery of the new myofibers. Hence, both Tnfsf14 and its receptors are expressed in regenerating myofibers during early stages of regeneration.

To probe the physiological function of Tnfsf14, we introduced Tnfsf14 shRNA at the time of muscle injury by co-injecting lentivirus expressing the shRNA with BaCl_2_ into TA muscles. Efficient knockdown of Tnfsf14 was confirmed by immunostaining (Figure 5b). Strikingly, muscle regeneration was markedly impaired by the knockdown, as evidenced by significantly fewer regenerating myofibers in the shTnfsf14-injected muscles compared with control shRNA-injected muscles during early regeneration (Figure 5c). Although some regenerating myofibers were present in the Tnfsf14 knockdown muscles, they were significantly smaller (Figure 5d). Interestingly, muscles with reduced Tnfsf14 expression also exhibited increased levels of cleaved PARP during the early phase of regeneration, suggesting higher levels of cell death (Supplementary Figure S4C). These results are in line with our *in vitro* data demonstrating that Tnfsf14 functions as pro-survival factor in myogenic cells (Figures 2a and c; Supplementary Figure S2A). Taken together, our observations provide strong evidence that Tnfsf14 is necessary for muscle regeneration post-injury.

### Tnfsf14 overexpression enhances skeletal muscle regeneration via activation of Akt

**Figure 6:**
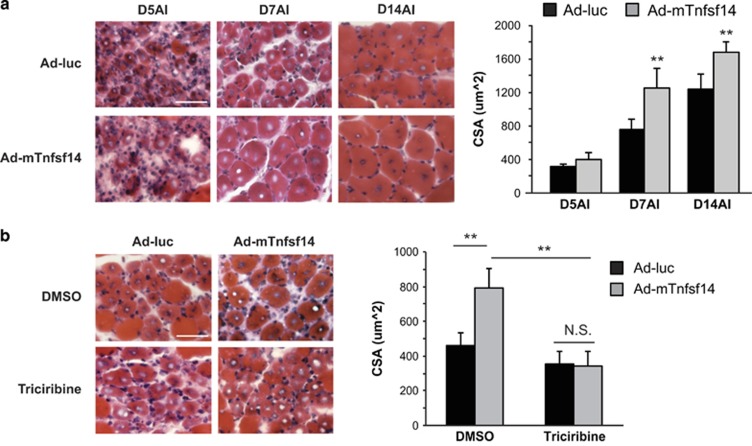
Local overexpression of Tnfsf14 enhances skeletal muscle regeneration in an Akt-dependent manner. (**a**) TA muscles were co-injected with BaCl_2_ and adenoviruses expressing mTnfsf14 (Ad-mTnfsf14) or luciferase (Ad-luc), and isolated on days 5, 7 and 14 AI. Upon cryosection, H&E staining was performed, and regenerating myofiber CSA was quantified. For each time point, five to seven mice were analyzed. (**b**) The procedure described in **a** was repeated, and the animals received daily intraperitoneal injection of 100 μl triciribine (0.26 *μ*g/μl) or 20% DMSO as a control, starting on the day of BaCl_2_ injection. For each condition, six mice were analyzed. All error bars represent S.D. of independent replicates. Paired two-tailed *t*-test was performed. **P<0.01. Scale bars: 50 *μ*m

Next, we examined the effect of Tnfsf14 overexpression on muscle regeneration. Adenovirus expressing mTnfsf14 was co-injected with BaCl_2_ into TA muscles. Remarkably, significantly larger regenerating myofibers were observed on day 7 and day 14 AI in mTnfsf14 adenovirus-injected muscles compared with muscles injected with a control adenovirus (Figure 6a). To test whether this regeneration-promoting effect of Tnfsf14 was through activation of Akt, we repeated this experiment in the presence of the Akt inhibitor triciribine. As shown in Figure 6b, daily injections of triciribine abrogated the effect of mTnfsf14 and the regenerating myofibers returned to a normal size. Therefore, overexpression of Tnfsf14 can enhance normal muscle regeneration post-injury in an Akt-dependent manner.

## Discussion

We have identified Tnfsf14 as a novel and essential regulator of skeletal myogenesis. Our findings reveal that Tnfsf14 functions as a pro-survival factor during myogenic differentiation, an effect mediated by its receptors HVEM and LT*β*R in an Akt-dependent manner. Furthermore, we show that Tnfsf14 is both essential for robust skeletal muscle regeneration, and capable of enhancing normal regeneration when administered exogenously. To date, only a handful of skeletal myocyte-secreted factors have been demonstrated to regulate myogenesis in a cell-autonomous manner, despite evidence that muscle cells are prolific cytokine secretors.^8, 10, 11^ We expect that future studies will reveal a large network of such cytokines involved in the differentiation and regeneration of skeletal muscle tissue.

By promoting myoblast survival, Tnfsf14 has a key role in ensuring a sufficient number of cells available for fusion during differentiation. Interestingly, the previously reported effects of Tnfsf14 on cell division, survival and death appear to be pleiotropic. Tnfsf14 has been described as pro-proliferation, anti-proliferation, pro-apoptosis and anti-apoptosis depending on the cellular contexts.^20, 21, 22^ We have found Akt to be a key mediator of Tnfsf14 action in muscle cells. Akt can prevent cell death by phosphorylation-mediated inactivation of a variety of important factors in apoptosis, including the Bcl2-related protein BAD, caspase-9 and the Forkhead transcription factor FKHRL1,^35, 36, 37^ as well as via activation of the major survival signaling molecule NF-κB.^38^ Although HVEM has been previously linked to Akt activation,^31^ the mechanism for this phenomenon is not fully understood. HVEM and LT*β*R are both TNFR superfamily members that lack the canonical TNFR death domain. Such TNFRs typically transduce signals by binding directly to TNF receptor-associated factors (TRAFs),^39^ and several TRAFs are believed to be involved in the activation of Akt via distinct mechanisms.^40^ HVEM and LT*β*R have been reported to bind TRAF1, 2, 3, 5, and TRAF2, 3, 5, respectively.^41, 42, 43, 44^ The exact mechanism(s) by which these two receptors activate Akt is certainly an area of potentially fruitful investigations in the future.

Of the reported muscle-secreted factors, insulin-like growth factor 1 and 2 (IGF1 and IGF2) have been well-established to support myoblast survival and differentiation.^45, 46^ *In vivo*, localized IGF1 overexpression promotes myofiber hypertrophy^47^ and accelerates muscle regeneration post-injury.^48, 49^ Our discovery of Tnfsf14's role expands the muscle cell's repertoire of secreted factors that regulate myoblast survival and muscle regeneration *in vivo*. It should be noted that many facets of IGF regulation in myoblasts, including survival, proliferation and myogenic gene expression, likely contribute to myogenic differentiation.^45^ In contrast, Tnfsf14 regulation of differentiation appears to be primarily through its survival function, as simply increasing cell number fully rescued fusion from Tnfsf14 deficiency (Figure 2d). It should also be noted that some degree of apoptosis during differentiation is believed to be necessary for myoblast fusion.^50^ It is conceivable that a network of cytokines governs the balance between apoptosis and survival during myoblast differentiation. Indeed, our functional screen of cytokines has revealed several known apoptosis-inducing factors as potential regulators.^12^ Future characterization of those candidates may be rewarding.

Throughout the process of skeletal muscle regeneration, there is a complex interplay between infiltrating immune cells, dying myofibers, activated muscle progenitor cells and regenerating fibers.^7^ It is important to note that the viruses we used to manipulate Tnfsf14 levels *in vivo* were not muscle cell specific. Thus, although muscle cells should be the prevailing cell type present during intramuscular injection, it is conceivable that other cell types (e.g., infiltrating macrophages) could also have been subjected to Tnfsf14 knockdown or overexpression during muscle injury. Tnfsf14 ablation specifically in muscle cells, such as in a muscle-specific knockout mouse, would allow more definitive studies probing the contribution of muscle-derived Tnfsf14 in adult muscle regeneration. Nevertheless, our study is the first to reveal Tnfsf14 expression in regenerating myofibers and the critical role of Tnfsf14 in myogenesis.

We observed that introduction of exogenous mTnfsf14 during muscle injury led to more robust muscle regeneration. Interestingly, this phenomenon does not translate to cultured cells *in vitro*. Although recombinant Tnfsf14 proteins rescue differentiation of C2C12 cells from Tnfsf14 knockdown, neither sTnfsf14 nor mTnfsf14 enhances differentiation in the presence of endogenous Tnfsf14. This discrepancy likely stems from the distinct environments of myoblasts *in vivo* *versus* *in vitro*. The injury caused by BaCl_2_ and subsequent inflammation *in vivo* likely introduces stronger pro-death signals than those found in differentiating C2C12 cells. It is possible that overexpression of Tnfsf14 has a more potent effect in tissues facing a greater challenge to survival. The fact that sTnfsf14 protects C2C12 cells from death induced by etoposide, a strong exogenous pro-death signal (Supplementary Figure S2A), may be further evidence for this hypothesis.

The effect of exogenous mTnfsf14 on enhancing muscle regeneration may have important implications in future development of therapeutics. Despite its myogenic potential, IGF1 is a growth factor that can promote cancer progression, and is thus a poor therapeutic tool in the context of cancer-associated cachexia.^15^ This is in stark contrast to Tnfsf14, which is a promising candidate for antitumor immunotherapy because of its ability both to recruit and activate T lymphocytes and dendritic cells to the tumor microenvironment, as well as to directly induce apoptosis of cancer cells.^20, 51^ Development of a cancer therapy that treats both the tumor and cancer 'side-effects' such as cachexia could simultaneously improve patient survival rates and quality of life. Tnfsf14 may be an attractive candidate to pursue in the search for such a multitasking treatment.

## Materials and Methods

### Antibodies and other reagents

Anti-MHC (MF20) and Ad5-luciferase adenovirus were obtained from the Developmental Studies Hybridoma Bank developed under the auspices of the NICHD, National Institutes of Health and maintained by the University of Iowa, Department of Biological Sciences. Anti-Tnfsf14 (C-20 or FL-240) and anti-HVEM (D-5) for western and immunohistochemistry were from Santa Cruz Biotechnology (Dallas, TX, USA). Anti-tubulin was from Abcam (Cambridge, MA, USA). Anti-LT*β*R was from Sigma-Aldrich (St. Louis, MO, USA). Anti-MyoD was from Novus Biologicals (Littleton, CO, USA). All other primary antibodies were from Cell Signaling Technology (Danvers, MA, USA). All secondary antibodies were from Jackson ImmunoResearch Laboratories, Inc. (West Grove, PA, USA). Gelatin and BrdU were from Sigma-Aldrich. Rat-tail collagen I was from Gibco, Thermo Fisher Scientific (Waltham, MA, USA). Recombinant sTnfsf14 was from PeproTech (Rocky Hill, NJ, USA). Triciribine was from EMD Millipore (Billerica, MA, USA). ELISA kit for detection of mouse sTnfsf14 was from Cloud Clone Corp. (Houston, TX, USA). The pCMV6-myristoylated-HA-Akt (c.a.-Akt) plasmid was previously reported.^52^

### Cell culture

C2C12 myoblasts were maintained in DMEM (4.5 g/l glucose) supplemented with 10% fetal bovine serum and 1% penicillin–streptomycin at 37 °C with 7.5% CO_2_. To induce differentiation, cells were plated on tissue culture plates coated with 0.2% gelatin and grown to 100% confluence before switching to differentiation medium (DMEM containing 2% horse serum). The cells were replenished with fresh differentiation medium daily for 3 days.

### Mouse primary myoblast isolation and differentiation

Primary myoblasts were isolated from 2- to 5-day-old FVB neonates as described previously,^53^ and maintained at low density on 50 *μ*g/ml collagen-coated tissue culture plates. Differentiation was induced at 70–80% cell density in differentiation medium for 2 days.

### Immunofluorescence microscopy and quantitative analysis of myocytes

C2C12 cells and primary myoblasts differentiated in 12-well plates were fixed and stained for MHC and with DAPI as previously described.^53^ When staining for mTnfsf14, the permeabilization step was omitted. Cells were examined with a Leica DMI 4000B fluorescence microscope (Leica Microsystems, Wetzlar, Germany). The fluorescent images were captured using a RETIGA EXi camera (QImaging, Surrey, BC, Canada), and analyzed with Image Pro Express software (Media Cybernetics, Rockville, MD, USA). The fusion index was calculated as the percentage of nuclei in myotubes with ⩾2 nuclei. Each data point was generated from quantifying all cells in five randomly chosen microscopic fields, totaling 2000–3500 nuclei for C2C12 cells and 300–900 nuclei for primary myoblasts.

### Lentivirus-mediated RNAi

shRNAs in the pLKO.1-puro vector were purchased from Sigma-Aldrich (MISSION TRC). Clone IDs are: shTnfsf14 #1, TRCN0000066398; shTnfsf14 #2, TRCN0000066400; shHVEM #1, TRCN0000065856; shHVEM #2, TRCN0000065857; shLT*β*R #1, TRCN0000065456; shLT*β*R #2: TRCN0000065457. A hairpin of scrambled sequence (shScramble) as a negative control and lentivirus packaging were previously described.^53^ Virally transduced C2C12 cells were selected in 3 *μ*g/ml puromycin for 2 days, followed by differentiation in media containing puromycin for 3 days. Virally transduced primary myoblasts were induced to differentiate without puromycin selection.

### Adenovirus-mediated Tnfsf14 overexpression

Adenovirus expressing Tnfsf14 (Ad-mTnfsf14; a kind gift from Dr. Yang-Xin Fu of the University of Chicago)^23^ and a control Ad5 adenovirus expressing luciferase (Ad-luc) were amplified in HEK AD293 cells. Briefly, a small amount of virus was used to infect AD293 cells. Two to 3 days post-transfection, the cells were detached from the plate via pipetting and centrifuged at 400x*g* for 5 min. Cells were then resuspended in PBS and subjected to four rounds of freeze/thaw cycles. The samples were centrifuged again at 10 000x*g* for 10 min, after which the supernatant was collected and used directly to infect C2C12 cells or primary myoblasts.

### Quantitative RT-PCR

C2C12 cells or regenerating muscles were lysed in Trizol (Invitrogen, Carlsbad, CA, USA), and RNA was isolated following the manufacturer's protocol. cDNA was synthesized from 1 *μ*g RNA using the RealMasterScript SuperMix cDNA synthesis kit (5Prime, Gaithersburg, MD, USA) following the manufacturer's protocol, followed by quantitative PCR on a StepOne Plus (Applied Biosystems, Foster City, CA, USA) using gene-specific primers. *β*-Actin was used as a reference to obtain the relative fold change for target samples using the comparative C_T_ method. Mouse *β*-actin primers: forward 5′-TTGCTGACAGGATGCAGAAG-3′ reverse 5′-ATCCACATCTGCTGGAAGGT-3′. Pre-validated mouse Tnfsf14 primers were purchased from Qiagen (QuantiTect Primer Assays, Hilden, Germany). Mouse HVEM and LT*β*R primers were previously published.^54, 55^

### Western blotting

Cells were lysed in SDS sample buffer with 10% *β*-mercaptoethanol. Proteins were resolved by SDS-PAGE and transferred onto polyvinylidene fluoride (PVDF) membrane (EMD Millipore Billerica, MA, USA) and incubated with various antibodies following the manufacturers' recommendations. Detection of horseradish peroxidase-conjugated secondary antibodies was performed with chemiluminescence solution (100 mM Tris-HCl, 0.009% H_2_O_2,_ 225 *μ*M coumaric acid, 1.25 mM luminol) and developed on X-ray films. Quantification of western blot band intensities was performed by densitometry of X-ray images using the ImageJ software (Bethesda, MD, USA).

### Cell proliferation and apoptosis assays

To measure proliferation of C2C12 cells, BrdU labeling was performed as previously described.^56^ To assess apoptosis, TUNEL assays were performed following the manufacturer's manual (Promega, Madison, WI, USA).

### Injury-induced muscle regeneration and manipulation of Tnfsf14 expression in mice

Male FVB mice aged 8–10 weeks were used in all the regeneration experiments. Muscle injury was induced by injection of BaCl_2_ (50 *μ*l of 1.2% w/v in saline) into TA muscles as previously described.^34^ On various days AI, the mice were killed, and the TA muscles were collected, followed by RNA extraction, or cryosection and staining. To knock down Tnfsf14, shTnfsf14 viruses (and shScramble as control) as described above but concentrated to 100x via ultracentrifugation were co-injected with BaCl_2_ into mouse hind limb TA muscles. To overexpress Tnfsf14, Ad-mTnfsf14 (Ad-luc as control) described above was co-injected with BaCl_2_ into TA muscles. The injected muscles were collected 5, 7 or 14 days AI and subjected to RNA isolation or cryosection.

### Muscle tissue cryosection, hematoxylin and eosin staining, and immunohistochemistry

TA muscles were isolated, frozen in liquid-nitrogen-cooled 2-methylbutane, and embedded in TBS tissue freezing medium (Thermo Fisher Scientific). Sections of 10-*μ*m thickness were obtained with a cryostat (Microm HM550; Thermo Fisher Scientific, Waltham, MA, USA) at −20 °C, placed on uncoated slides, and stained with hematoxylin and eosin (H&E). Separately, the sections were fixed by 1.5% paraformaldehyde, incubated with anti-Tnfsf14, HVEM, LTβR or cleaved PARP antibody, followed by incubation with Alexa-conjugated secondary antibodies and DAPI. Imaging was performed with a fluorescence microscope (DMI 4000B; Leica) with a 20 × dry objective (Fluotar, numerical aperture 0.4; Leica). The bright field and fluorescence images were captured at 24 bit and 8 bit, respectively, at room temperature using a camera (RETIGA EXi; Q-Imaging, Surrey, BC, Canada) equipped with Image Pro Express software (Media Cybernetics). The images were then processed in Photoshop CS5 (Adobe), where brightness and contrast were adjusted. Fluorescence images were pseudo-colored and adjusted, when necessary, by identical parameters for all samples in the same experiment. A total area of 464 000 μm^2^ from the degenerated regions of each TA muscle was scored for centrally nucleated regenerating myofiber numbers and their cross-sectional area (CSA). The CSA of a minimum of 100 myofibers was measured to generate each data point.

### Statistics

All data shown are representative results of at least three independent experiments, or *n*⩾5 for animal experiments. All quantitative data are presented as mean±S.D. Whenever necessary, statistical significance of the data comparison was analyzed by performing one-sample or paired two-tailed *t*-tests. *P<0.05; **P<0.01.

### Study approval

All animal experiments in this study followed protocols approved by the Animal Care and Use Committee at the University of Illinois at Urbana-Champaign.

## Abbreviations

BaCl_2_: barium chloride
BrdU: bromodeoxyuridine
HVEM: herpes virus entry mediator
IGF: insulin-like growth factor
IL-1: interleukin-1
IL-6: interleukin-6
LT*β*R: lymphotoxin *β* receptor
MHC: myosin heavy chain
TA: tibialis anterior
TNF*α*: tumor necrosis factor *α*
Tnfsf14: tumor necrosis factor superfamily member 14 (Ad-mTnfsf14, adenovirus expressing membrane-bound Tnfsf14
sTnfsf14: soluble Tnfsf14
TRAF: tumor necrosis factor receptor-associated factor
