# Supplementary material for: Myocyte-derived Tnfsf14 is a survival factor necessary for myoblast differentiation and skeletal muscle regeneration
Source: Cell Death Dis. 2015 Dec 31;6(12):e2026–. doi: 10.1038/cddis.2015.375 (PMC4720906; doi:10.1038/cddis.2015.375)
Supplement: Supplementary Figures [file cddis2015375x1.pdf]

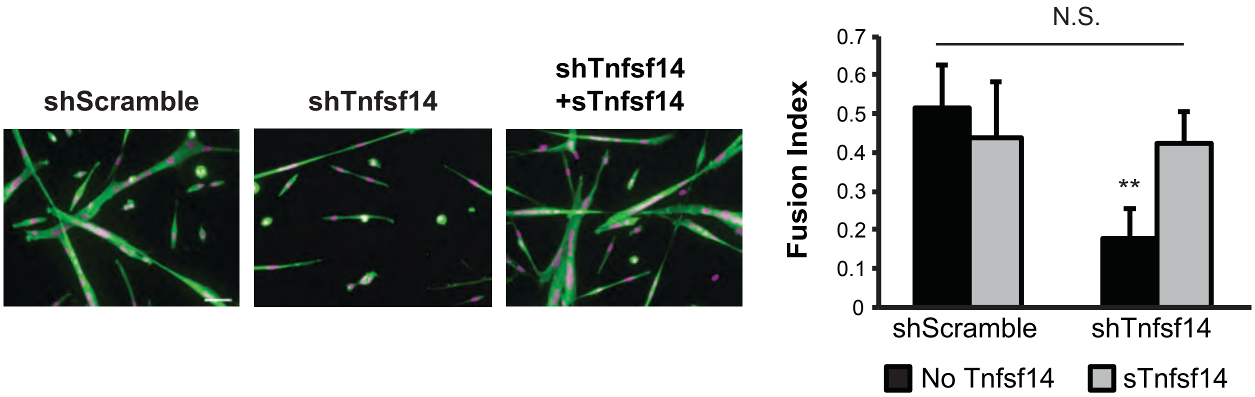

Fig. S1. Tnfsf14 is necessary for primary myoblast differentiation
Mouse primary myoblasts were infected with shRNA lentiviruses and differentiated for 48 hrs with or without 50 ng/mL sTnfsf14, followed by staining for MHC (green) and with DAPI (red). The fusion index was quantified (n=3). Error bars represent SD of independent replicates. Scale bar: 50 μm.

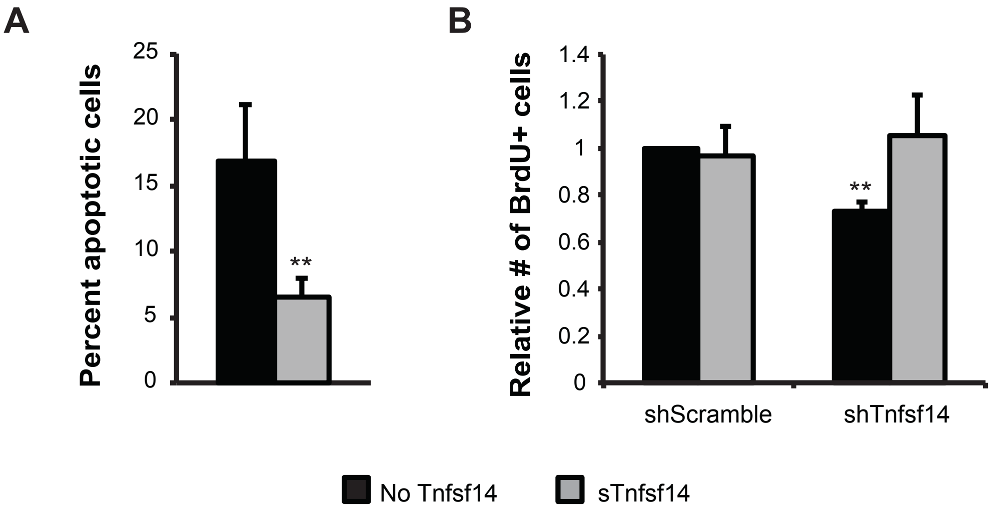

Fig. S2. Tnfsf14 suppresses apoptosis and promotes proliferation of C2C12 cells
(A) Proliferating C2C12 cells were exposed to 10 μM etoposide for 24 hours in the presence or absence of 25 ng/mL recombinant sTnfsf14. TUNEL assays were performed to assess the percentage of apoptotic cells (n=5).
(B) C2C12 cells were treated as in (A) and subjected to BrdU labeling to assess cell proliferation (n=4).
All error bars represent SD of independent replicates.

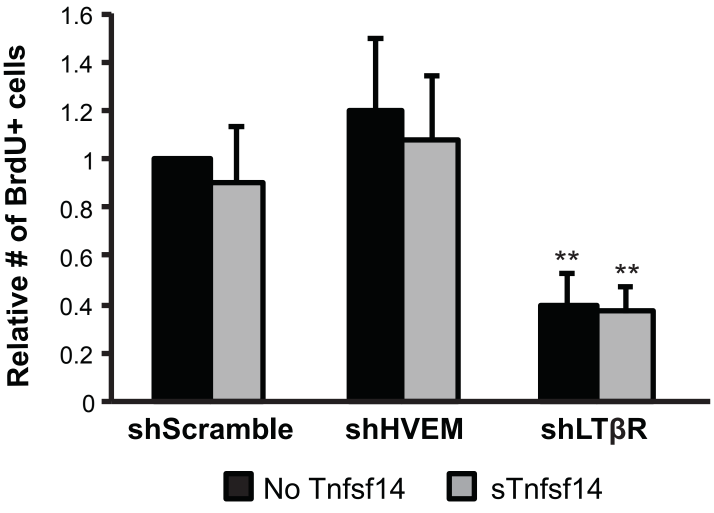

Fig. S3. Tnfsf14 receptors in cell proliferation
C2C12 cells were infected with shRNA lentiviruses as indicated and differentiated for 24 hrs in the presence or absence of 25 ng/mL recombinant sTnfsf14, followed by BrdU labeling to assess cell proliferation (n=4). All error bars represent SD of independent replicates.

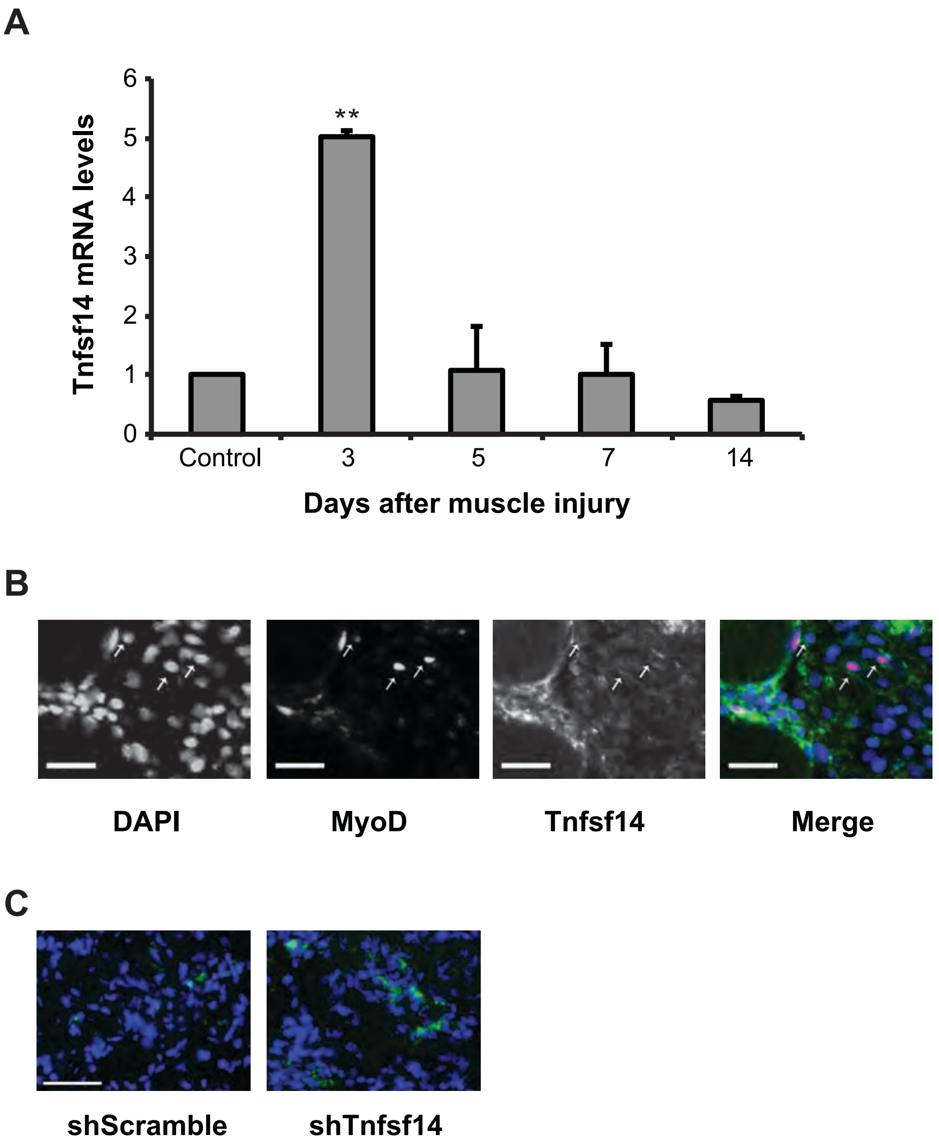

Fig. S4. Tnfsf14 expression during muscle regeneration
(A) TA muscles were injured by $BaCl_2$ injection, and isolated on days 3, 5, 7, and 14 after injury (AI). RNA was isolated and subjected to qRT-PCR assays to measure relative levels of Tnfsf14 mRNA (n=3). Error bars represent SD of independent replicates. One-sample 2-tailed t test was performed to compare data at each time point to control. **$P<0.01$.
(B) TA muscles were injured by $BaCl_2$ injection, and isolated on day 3 after injury (AI). Upon cryosection, immunofluorescence staining for Tnfsf14 (green), MyoD (red), and DAPI (blue) was performed (n=3). Scale bar: 25 μm. Arrows indicate some of the Tnfsf14+/MyoD+ cells.
(C) TA muscles were co-injected with $BaCl_2$ and shRNA viruses, and isolated on day 3 AI. Upon cryosection, immunofluorescence staining for cleaved PARP (green) and DAPI (blue) was performed (n=3). Scale bar: 50 μm.
